# Development and Symbiosis Establishment in the Cnidarian Endosymbiosis Model *Aiptasia* sp.

**DOI:** 10.1038/srep19867

**Published:** 2016-01-25

**Authors:** Madeline Bucher, Iliona Wolfowicz, Philipp A. Voss, Elizabeth A. Hambleton, Annika Guse

**Affiliations:** 1 Centre for Organismal Studies (COS), Heidelberg University, Heidelberg 69120, Germany; 2 Graduate Program in Areas of Basic and Applied Biology (GABBA), University of Porto, Porto 4200-465, Portugal

## Abstract

Symbiosis between photosynthetic algae and heterotrophic organisms is widespread. One prominent example of high ecological relevance is the endosymbiosis between dinoflagellate algae of the genus *Symbiodinium* and reef-building corals, which typically acquire symbionts anew each generation during larval stages. The tropical sea anemone *Aiptasia* sp. is a laboratory model system for this endosymbiosis and, similar to corals, produces non-symbiotic larvae that establish symbiosis by phagocytosing *Symbiodinium* from the environment into the endoderm. Here we generate the first overview of *Aiptasia* embryogenesis and larval development and establish *in situ* hybridization to analyze expression patterns of key early developmental regulators. Next, we quantify morphological changes in developing larvae and find a substantial enlargement of the gastric cavity over time. Symbiont acquisition starts soon after mouth formation and symbionts occupy a major portion of the host cell in which they reside. During the first 14 days of development, infection efficiency remains constant while in contrast, localization of phagocytosed symbionts changes, indicating that the occurrence of functional phagocytosing cells may be developmentally regulated. Taken together, here we provide the essential framework to further develop *Aiptasia* as a model system for the analysis of symbiosis establishment in cnidarian larvae at the molecular level.

Coral reef ecosystems, of high ecological and economic relevance, strictly depend upon a functional symbiosis between corals and dinoflagellates (genus *Symbiodinium*), which reside inside host endodermal cells and provide most of the host’s nutrition via transferred photosynthates, receiving inorganic nutrients and shelter in return^1^. The genus *Symbiodinium* comprises hundreds of strains^2^, with reef-building coral taxa establishing relationships with some strains but not others; such symbiosis specificity produces physiological consequences, such as adaptation to certain environmental conditions^3^,^4^,^5^. Most scleractinian corals (>75%) transmit symbionts horizontally, thereby re-establishing symbiosis each generation and allowing symbiont-host combinations that differ from those of the parents^6^,^7^.

In anthozoans that undergo horizontal symbiont transmission, non-symbiont-containing embryos develop into planula larvae (hereafter larvae), a ciliated postgastrula stage that, like the adult morphology, is diploblastic with defined endodermal and ectodermal tissue layers connected by the mesoglea^8^,^9^,^10^. Larvae of the majority of reef-building corals demonstrably have the capacity to acquire symbionts from the surrounding marine environment^11^,^12^,^13^,^14^,^15^. One horizontally transmitting coral species (*Fungia scutaria*) has also been shown to integrate symbionts during embryogenesis, i.e. before mouth formation^16^. However, the majority of corals appear to require an open blastopore leading into a developed gastric cavity for symbiont uptake and phagocytosis^15^. Harii and colleagues documented an increase in gastric cavity size as coral larvae age, and postulated that this enlargement may be widespread in corals and accompanied by the occurrence of well-developed, ciliated gastrodermal cells that may enhance acquisition of symbionts by late developmental stages^15^.

Despite the critical importance of symbiont acquisition in coral larvae, it is not yet clear whether the larval endoderm consists of different cell types, some of which may be specialized phagocytes, and whether the occurrence of such cell types is developmentally regulated. Also unknown is whether and how symbiont phagocytosis alters the morphology and physiology of the host endoderm at the single-cell level and/or the broader tissue context. This lack of knowledge is largely because the systematic study of the detailed cellular and molecular events leading to symbiosis establishment is hindered by the once-annual spawning of most reef-building corals^7^,^17^. Much of our understanding of cnidarian development and larval physiology comes from well developed model systems such as *Nematostella vectensis*, *Clytia hemisphaerica*, and *Hydractinia echinata*; however, the former is non-symbiotic and the latter two are medusozoans whose larvae therefore differ considerably from corals (e.g. lack of an open blastopore and accompanying pharyngeal region lined by the endodermal epithelia)^18^,^19^. Thus, a tractable laboratory model of symbiont acquisition in larvae has heretofore been largely absent.

To address this limitation, a practicable symbiotic laboratory model has been developed with the sea anemone *Aiptasia* sp., an anthozoan that forms relationships with the same types of *Symbiodinium* as corals^20^,^21^ and likewise exhibits horizontal transmission of these symbionts through production of non-symbiotic larvae^22^. Most importantly, spawning can be induced efficiently in *Aiptasia* under laboratory conditions, providing regular access to abundant larvae for experimentation^23^. Such experimentation in *Aiptasia* is particularly exploitable because of the cellular and molecular resources already generated for the system, including the *Aiptasia* genome^24^ and several transcriptomes^25^,^26^ and corresponding transcriptomic/genomic resources for several *Symbiodinium* strains^27^,^28^,^29^.

With the *Aiptasia* model system in place, we sought to address key questions of how early development of the anthozoan larva contributes to symbiosis establishment. Here we provide for the first time an analysis of the basic features of *Aiptasia* development from embryogenesis to late larval stages, including how larval morphology changes over time. Specifically, we find that the gastric cavity enlarges over time, predominantly through remodeling of the endodermal tissue. By co-incubating larvae with a compatible *Symbiodinium* strain, we find that symbiont uptake starts after mouth formation and that uptake efficiency remains constant for two weeks. Symbionts are integrated into the host cells and appear to locally alter endodermal morphology. Interestingly, the spatial distribution of symbiont uptake within the endoderm changes as larvae age, indicating that the occurrence of symbiont-phagocytosing cells may change over time. Taken together, we provide a foundational platform for studies of the specific molecular and cellular processes of symbiont phagocytosis and other events critical to symbiosis establishment.

## Results

### Characterization of embryonic and larval development in *Aiptasia*

**Figure 1:**
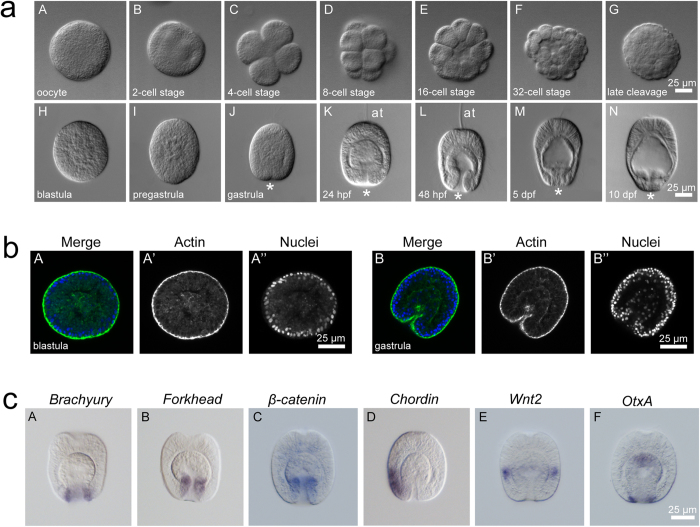
Development of *Aiptasia*. (**a**) Overview of *Aiptasia* embryonic and larval development using differential interference contrast (DIC) microscopy. *indicates the blastopore; hpf = hours post fertilization; dpf = days post fertilization. (**b**) Representative confocal microscopy images of *Aiptasia* blastula (A–A”) and gastrula (B–B”). The left panels (A and B) show merged images of Hoechst-stained nuclei (blue) and phalloidin-stained F-actin (green), the middle panels (A’ and B’) only actin, and the right panels (A” and B”) only nuclei. (**c**) Gene expression patterns of key classical developmental regulators in *Aiptasia* larvae ∼24 hpf using *in situ* hybridization.

A prerequisite of symbiosis studies in larval stages is an initial characterization of *Aiptasia* embryogenesis under standard conditions, which has heretofore not been reported. To generate such an overview, we used differential interference contrast (DIC) microscopy to image development from the unfertilized egg to planula larvae 10 days post fertilization (dpf) (Fig. 1a). After fertilization of eggs (diameter 85.8 +/− 5 μm [*n* = 51]), two meridional and one equatorial cleavages produce sister cells of similar size with very stereotypic 4-cell, 8-cell and 16-cell stages (Fig. 1a, C–E). For the 16- and 32-cell stages, the formation of a small blastocoel is observed (Fig. 1a, E–F). Similar to *Nematostella*^30^, we were unable to observe polar bodies, fertilization membranes, or prominent 2-cell stages (Fig. 1a, A–C). In the ciliated blastula, which begins to rotate, the nuclei are localized to the periphery (Fig. 1a, H; b, A–A”). Before gastrulation (which appears to occur predominantly via invagination), the blastula starts to elongate (Fig. 1a, I; b, B–B”) and an apparent blastopore forms (Fig. 1a, J). At 24 hours post fertilization (hpf), two germ layers can be distinguished, with the endoderm filling the larval cavity; concurrently, at the aboral pole the apical ciliary tuft forms, which may be involved in settlement and metamorphosis (Fig. 1a, K)^31^,^32^. At 48 hpf we observed typical features of anthozoan planula larvae, including a clear oral-aboral axis with a prominent blastopore at the oral pole and a well-developed apical tuft at the aboral pole. Defined endodermal and ectodermal tissue layers form, separated by the mesoglea, and the first mature nematocytes are distinguishable within the aboral ectoderm (Fig. 1a, L). As larvae mature, the endoderm appears to get thinner and the gastric cavity more spacious (Fig. 1a, M–N). Under laboratory conditions, larvae can be maintained for approximately 30–40 days before they die; presumably, in nature larvae find a proper substrate for settlement during this time, but settlement has not been achieved in the laboratory to date (see Discussion).

To generate a molecular snapshot of early development in *Aiptasia*, we used *in situ* hybridization to determine the spatial expression of classical developmental markers involved in tissue specification and body axis formation in gastrulating embryos (Fig. 1c). Genes encoding the transcription factors *Brachyury* and *Forkhead*, as well as *β-catenin* are expressed at the blastopore (Fig. 1c, A–C), whereas *Chordin* is expressed only on one side in the anterior ectoderm (Fig. 1c, D). The *Wnt* most aborally located, *Wnt2*, forms a distinct domain in the ectoderm and *OtxA* is expectedly found throughout the endodermal tissue and around the blastopore (Fig. 1c, E–F). All six markers faithfully replicated localization patterns seen in *Nematostella* at comparable developmental stages (late gastrula to planula larva stages)^10^,^33^,^34^,^35^,^36^,^37^,^38^, indicating that *Aiptasia* follows a conserved anthozoan developmental program.

### Morphological changes in the symbiosis-relevant endoderm during development

**Figure 2:**
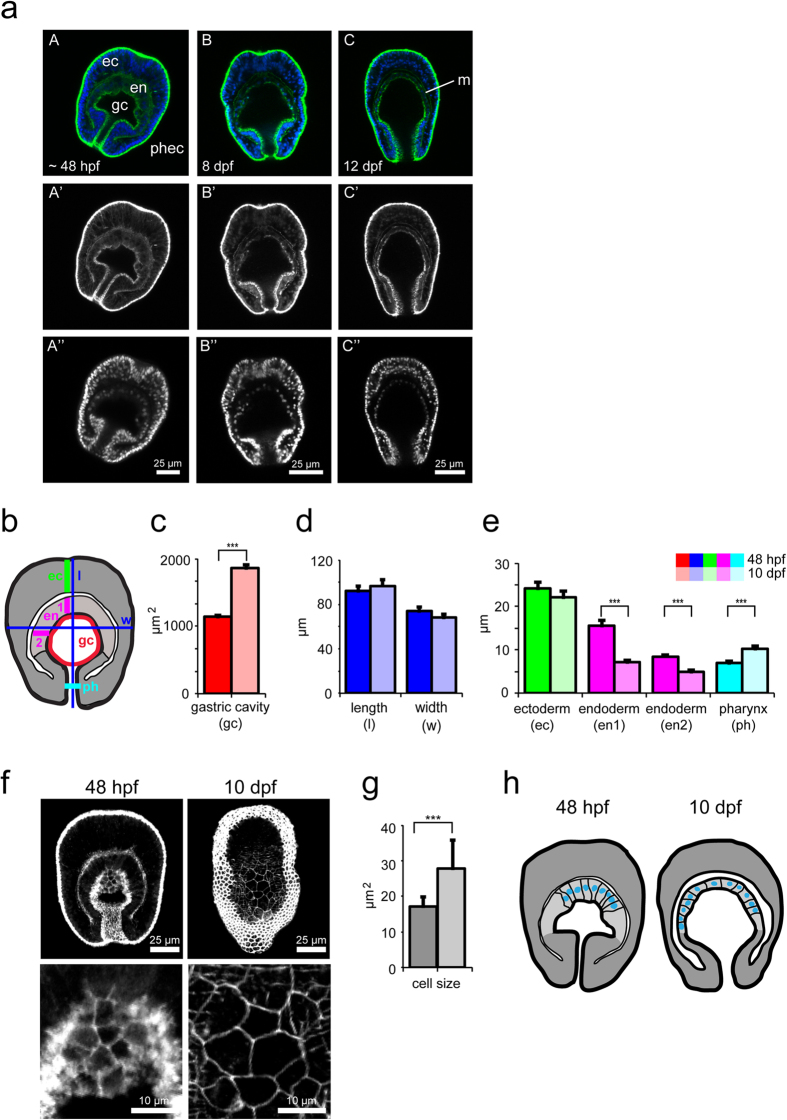
Morphological changes of the larval endoderm during development. (**a**) Representative confocal microscopy images of *Aiptasia* larvae 48 hpf (A–A”), 8 dpf (B–B”), and 12 dpf (C–C”). The upper row (A–C) shows merged images of Hoechst-stained nuclei (blue) and phalloidin-stained F-actin (green), the middle row (A’–C’) only actin, and the lower row (A”–C”) only nuclei. The endoderm (en), ectoderm (ec), gastric cavity (gc), pharyngeal ectoderm (phec), and mesoglea (m) are indicated. (**b**) Schematic of larva with colored lines indicating positions of measurement of morphological features in (**c–e**). *n* = 23 for larvae 48 hpf and *n* = 22 for larvae 10 dpf. (**c**) Quantification of change in gastric cavity area between larvae 48 hpf and 10 dpf. Error bars are SEM, ***p < 0.001 as determined by Student’s *t*-test for unpaired data. (**d**) Quantification of change in larval length and width between larvae 48 hpf and 10 dpf. Error bars are SEM. (**e**) Quantification of change in thickness of the ectoderm (ec), endoderm (en1, en2), and pharyngeal width (ph) between larvae 48 hpf and 10 dpf. Error bars are SEM, ***p < 0.001 as determined by Student’s *t*-test for unpaired data. (**f**) Representative confocal microscopy images of *Aiptasia* larvae 48 hpf and 10 dpf showing phalloidin-stained F-actin to mark the cell outlines. Each image comprises z-projections of multiple planes of the endoderm. Below are corresponding higher-magnification images of endodermal cells. (**g**) Quantification of endodermal cell sizes of larvae 48 hpf and 10 dpf from images as shown in (**f**). Error bars are SEM, ***p < 0.001 as determined by Student’s *t*-test for unpaired data, *n* = 56 cells for larvae 48 hpf (5 larvae) and *n* = 82 cells for larvae 10 dpf (5 larvae). (**h**) Schematic of larvae summarizing morphological changes: younger larvae (48 hpf) have a small gastric cavity, a thick endoderm with columnar cells, and a pronounced pharyngeal ectoderm when compared to older larvae (10 dpf), which have a bigger gastric cavity, flattened endodermal cells, smaller pharyngeal ectoderm, and more pronounced mesoglea.

We next sought to characterize changes in larval tissue morphology over the course of development, with particular attention to features that may influence the establishment of symbiosis. To do so, we examined larval tissue structure in detail by using confocal microscopy to visualize nuclei (Hoechst staining) and cell boundaries (phalloidin staining of F-actin) in larvae of different ages. Larvae 48 hpf already have clearly defined tissue layers: the endoderm and the ectoderm, which also lines the prominent pharynx connecting the mouth to the gastric cavity (Fig. 2a, A). As larvae transition to middle age (8 and 12 dpf), the pharyngeal ectoderm noticeably shrinks and the gastric cavity appears to become considerably larger (Fig. 2a, B–C).

Symbionts are taken up from the environment into the gastric cavity, where they are then phagocytosed by host endodermal cells^12^,^14^,^39^. We therefore quantified the enlargement of the gastric cavity over larval aging. By measuring the area of the gastric cavity in one medial plane as a proxy (see schematic in Fig. 2b), we find that the gastric cavity is approximately 2.5 fold larger in larvae 10 dpf (~1500 μm^2^) than in larvae 48 hpf (~600 μm^2^) (Fig. 2c). Quantification of larval length and width shows that neither metric differs significantly between larvae 48 hpf and 10 dpf, demonstrating that the gastric cavity enlargement is not simply because the whole larva enlarges, as it rather remains relatively constant in size (Fig. 2b,d).

To identify the cause of the increase in gastric cavity size, we measured ecto- and endodermal thickness as well as the width of the pharynx lined by the pharyngeal ectoderm (Fig. 2b,e). The thickness of the ectoderm at the aboral region remains constant between these two larval stages. In contrast, the endodermal thickness changes substantially during this time: while the aboral endoderm is ~15 μm thick in larvae 48 hpf, it is reduced nearly 50% to ~7 μm thick in larvae 10 dpf. Likewise, the endoderm lining the sides of the gastric cavity decreases from ~9 μm to ~5 μm. Moreover, the width of the pharynx increases approximately 30%, from ~7 μm to ~10 μm (Fig. 2e). To observe these morphological changes at the cellular level, we measured the areas of the apical faces of the endodermal cells in larvae 48 hpf and 10 dpf (Fig. 2f). We find that in larvae 48 hpf, the areas of endodermal cells are substantially smaller (~17 μm^2^) than those in larvae 10 dpf (~28 μm^2^) (Fig. 2g). However, the cell areas in older larvae range from less than 10 μm^2^ to more than 50 μm^2^, whereas the cells in younger larvae are more uniform (Fig. 2f,g).

Taken together, the data above indicate that over time, the larval endodermal architecture changes from long, columnar cells with uniform cell surface areas to relatively flatter cells with more variable cell surface areas. Together with the widening of the pharynx, these morphological changes allow the gastric cavity to increase in size as the larvae age. These changes are summarized schematically in Fig. 2h.

### Developmental time window of symbiosis establishment and consequent endodermal remodeling

**Figure 3:**
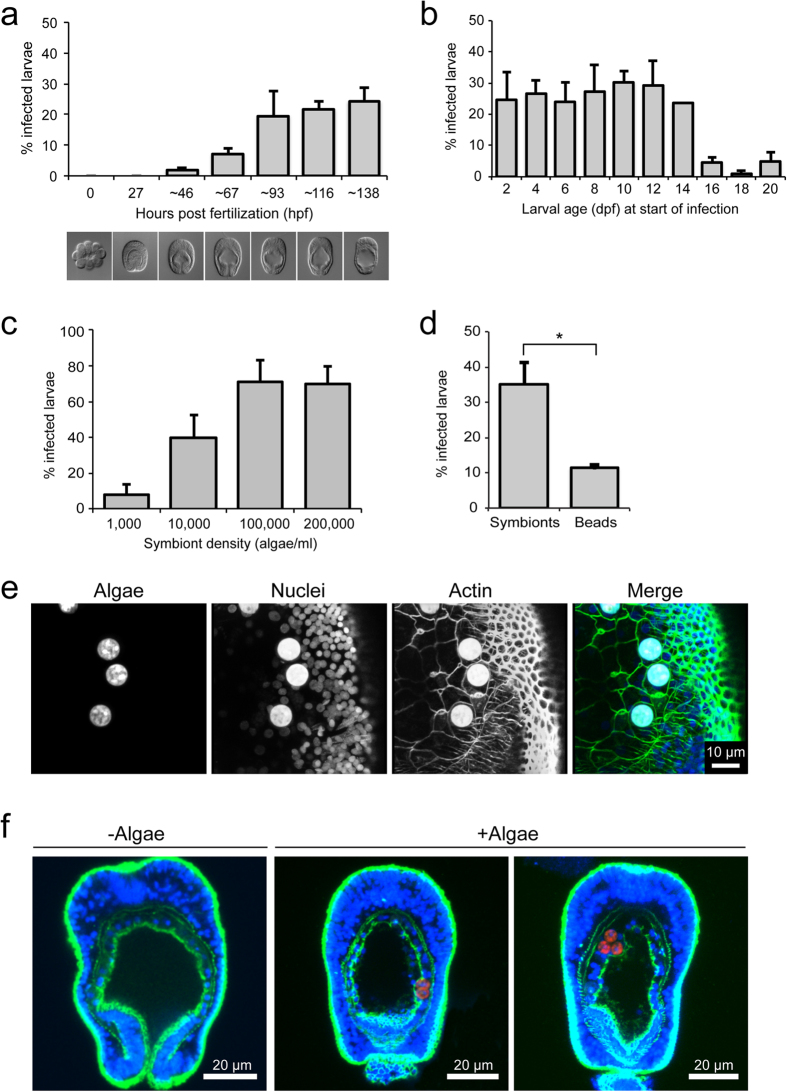
Symbiosis establishment during larval development. (**a**) Quantification of symbiont uptake efficiencies in *Aiptasia* embryos and early planula larvae: early (<32-cell-stage) embryos were exposed to a constant environmental supply of *Symbiodinium* strain SSB01^21^ (10,000 algae/ml) and subsets were sampled at the times indicated (hpf) to assess infection efficiency. Representative DIC images are shown below each timepoint. Error bars are SEM, *n* = 3 replicate experiments. (**b**) Quantification of symbiont uptake efficiencies for larvae between 2 and 20 dpf: larvae at the ages indicated (dpf) were incubated with *Symbiodinium* strain SSB01 (10,000 algae/ml) for four days, after which infection efficiency was assessed. Error bars are SEM, *n* = 3 replicate experiments. (**c**) Quantification of symbiont uptake efficiencies after four days exposure for larvae 6–7 dpf at increasing algal concentrations. Error bars are SEM, *n* = 3 replicate experiments. (**d**) Quantification of comparison of uptake efficiency between SSB01 algae and inert fluorescent beads for larvae 4 dpf after four days exposure. Error bars are SEM, ***p < 0.001 as determined by Student’s *t*-test for unpaired data, *n* = 3 replicate experiments. (**e**) Representative fluorescence microscopy images of phagocytosed symbionts in endodermal cells of larvae 8 dpf. Hoechst-stained nuclei are shown in blue, phalloidin-stained F-actin to mark cell outlines in green, and endogenous autofluorescence of algal chlorophyll in red. Note that algae exhibit strong autofluorescence in all channels. (**f**) Representative confocal microscopy images of larvae 10 dpf with or without symbionts. Fluorescence channels are as in (**e**).

The observed broad-scale changes in the symbiosis-relevant endoderm led us to determine the period in development in which naturally non-symbiotic *Aiptasia* larvae acquire symbionts from the environment. To test when larvae are first capable of symbiont acquisition, we added a constant environmental supply of a compatible *Symbiodinium* strain (SSB01^21^) at 10,000 algal cells/ml to developing embryos at the 4-, 8-, and 16-cell stages and fixed a subset of embryos at different time-points early in development to measure infection efficiencies (Fig. 3a). Algae were first detected in larvae between 1 and 2 dpf (Fig. 3a); by this time, the mouth had formed and the endodermal and ectodermal tissue layers were clearly distinguishable. However, infection was very low (~3%), suggesting only a recent ability of the larvae to acquire algae, likely largely due to recent mouth formation. Infection increased over time to ~20–25% as larvae matured (we did not distinguish between algae inside the gastric cavity and those integrated into the endodermal tissue) (Fig. 3a).

We next analyzed symbiont uptake efficiencies in older larvae by monitoring over longer periods of time. To this end, we incubated larvae with a constant environmental supply of *Symbiodinium* strain SSB01 (again 10,000 algae/ml) for incremental four-day windows, beginning with larvae 48 hpf, and then measured the infection efficiencies at the end of each of these four-day windows. Between 2 and 14 dpf, ~25–30% of *Aiptasia* larvae take up symbionts from the environment; however, at 15–16 dpf, symbiont uptake by larvae is drastically decreased (Fig. 3b). Taken together, these experiments indicate that larval competency for symbiosis establishment peaks in middle-aged larvae, with young larvae (48 hpf) just gaining competency and older larvae (over 16 dpf) losing competency.

Through similar experiments as above (larvae 6–7 dpf exposed to *Symbiodinium* for four days), we find that symbiont uptake efficiency increases with algal concentration: at 100,000 algae/ml, more larvae (>70%) take up symbionts than at 10,000 algae/ml (∼40%) and at 1,000 algae/ml (∼10%). However, infection efficiency does not further increase at 200,000 algae/ml, indicating saturation (Fig. 3c). After four days exposure at non-saturating algal concentrations (10,000 algae/ml), SSB01 symbionts are taken up by larvae 4 dpf more efficiently than inert fluorescent beads, a proxy for food particles^14^ of similar size (∼7 μm) (Fig. 3d). These data indicate that *Aiptasia* larvae may distinguish between SSB01 algae and inert particles, preferentially taking up the former, and that this uptake may depend on the frequency of encounters between larvae and algae.

To visualize the cellular architecture of symbionts housed within larval host cells during symbiosis establishment, we again used confocal microscopy to visualize nuclei (Hoechst staining), cell boundaries (phalloidin staining of F-actin), and symbionts (endogenous algal chlorophyll autofluorescence). Anthozoan endodermal cells have been shown to be rather small (i.e. 10 μm × 25 μm) when compared to symbiont sizes (∼7–10 μm in diameter), yet they typically house one or two (and sometimes up to twelve) symbiont cells^21^,^40^,^41^. As such, we observe that phagocytosed symbionts occupy a major portion of their host cells in *Aiptasia* larvae (Fig. 3e). When observed at the tissue level, this tight cell-within-cell arrangement and its consequent effects on endodermal organization become more apparent: the endodermal tissue containing symbionts consistently bulges out into the gastric cavity (Fig. 3f).

### Changes in endodermal localization of symbionts during development

**Figure 4:**
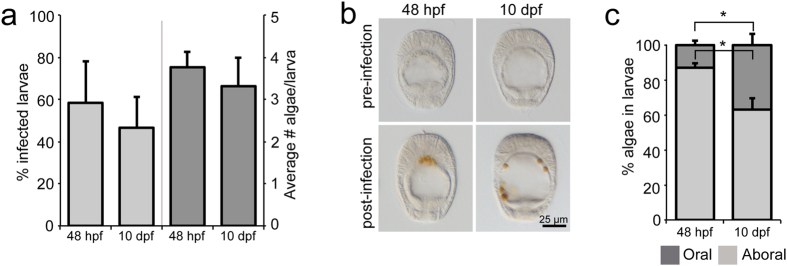
Change in localization of symbiosis establishment during development. (**a–c**) After 24 h exposure to symbionts (100,000 algal cells/ml), larvae 48 hpf and 10 dpf were scored for infection efficiency and average number of algal cells per larva. *n* = 3 replicate experiments (**a**). Additionally, the localization of the algal cells in the aboral or oral endoderm in each larva was recorded: representative DIC microscopy images are shown in (**b**) and quantification in (**c**). *n* = 3 replicate experiments, ≥30 larvae per experiment. Error bars are SEM, *p < 0.05 as determined by Student’s *t*-test for unpaired data.

We then sought to connect the observed endodermal changes during regular development with the endodermal remodeling seen during symbiosis establishment, in order to address the question of how these changes may affect symbiosis establishment. To this end, we incubated non-symbiotic larvae 48 hpf or 10 dpf with a constant supply of *Symbiodinium* strain SSB01 algae at 100,000 algae/ml for 24 h, after which larvae were fixed and three parameters were measured: infection efficiency (measured in percent of larvae containing one or more algal cells), exact number of algal cells per larva, and localization of the algae within the larvae. We found that symbiont uptake efficiency does not differ markedly between larvae 48 hpf and 10 dpf, with approximately 50%–60% of larvae containing algae (Fig. 4a). Likewise, the average number of algae in each larva remained essentially constant between the younger and older larvae, with most larvae containing three or four algal cells (Fig. 4a).

In contrast, when we analyzed how symbiont phagocytosis is spatially regulated within the endodermal tissue, we observed a striking difference between younger and older larvae. In younger larvae, algal cells are located primarily in the aboral endoderm, whereas in older larvae, algal cells are distributed throughout both the aboral and oral regions of the endoderm (Fig. 4b). Quantification of this phenomenon further emphasized this difference: nearly 90% of the algal cells in larvae 48 hpf were in the aboral endoderm, whereas in larvae 10 dpf, only around 60% of algal cells were in the aboral endoderm, with the remainder appearing in the oral endoderm (Fig. 4c).

## Discussion

Here we provide the first overview of *Aiptasia* embryogenesis and key morphological features of *Aiptasia* planula larvae as well as a quantification of vital aspects of symbiosis establishment in relation to *Aiptasia* development. Similar to many anthozoans, *Aiptasia* embryos undergo stereotypic and holoblastic cleavages to form a ciliated blastula before gastrulation gives rise to the characteristic diploblastic anthozoan planula larva with an open blastopore, defined gastric cavity, and apical tuft^8^,^31^. However, we also found differences between embryonic/larval development of *Aiptasia* and that of other anthozoan systems such as *Nematostella* or corals. For example, we never observe prawn chip stages preceding blastula formation^30^,^42^. Neither do we observe mesentery formation within *Aiptasia* larvae as reported for late larval stages in *Nematostella*^43^,^44^. This difference is likely due to the fact that *Nematostella* larvae spontaneously initiate metamorphosis and settlement under laboratory conditions, while *Aiptasia* larvae have not yet been reported to metamorphose or settle spontaneously under laboratory conditions. This suggests that *Aiptasia* larvae, similar to corals, may need specific cues that induce neuropeptide expression to exit larval stages and proceed with development into the polyp stage^45^. As mesentery formation might mark the beginning of metamorphosis, we therefore expect it to be observed in *Aiptasia* larvae once these metamorphosis cues have been identified. The identification of these specific cues to promote progression of *Aiptasia* development, thus closing the life cycle in the laboratory, is an important step for the *Aiptasia* laboratory model system. More broadly, the overview of *Aiptasia* early development together with the *in situ* hybridization protocol to analyze gene expression patterns presented here now opens the door for comparative molecular analyses of *Aiptasia* development to that of other cnidarian larvae, especially *Nematostella*, to dissect the similarities and differences between two distinct representatives of anthozoan larval forms.

To study phagocytosis of symbionts by endodermal cells during horizontal transmission at the molecular level, detailed knowledge of the temporal and spatial regulation of symbiont uptake in *Aiptasia* larvae is essential. We found that *Aiptasia* does not take up symbionts during embryogenesis as suggested for solitary non-colonial corals (e.g. *Fungia scutaria*)^16^ but rather resembles the majority of colonial coral species tested to date in that the formation of an open mouth and a developed gastric cavity are prerequisites for symbiont uptake^15^. *Aiptasia* larvae phagocytose symbionts with similar efficiency between 48 hpf and ∼16 dpf, after which uptake decreases in these conditions. However, we currently cannot distinguish whether this is an inherent characteristic of larvae or whether it was because no food was provided in these experiments, possibly causing starvation and the consequent inability to phagocytose symbionts. Indeed, many anthozoan larvae appear to be planktotrophic^8^,^11^ and recent experiments showed that *Nematostella* larvae may assimilate certain types of dissolved organic matter (DOM)^46^. Thus, the time window for symbiont uptake in *Aiptasia* larvae may even be larger in nature, where food can be assimilated from the environment. It will therefore be interesting to test whether food supply can extend the window of symbiont uptake competency in *Aiptasia* larvae in the future. Moreover, it will be important to analyze if and how energy derived through food uptake and photosynthetically active symbionts affects the survival of *Aiptasia* larvae. Both may be important factors in extending larval lifetime and hence dispersal, both of which have profound effects on species biogeography. This is particularly important for sessile symbiotic anthozoans, including *Aiptasia* and corals, which depend upon favorable environmental conditions (e.g. temperature and light) for a thriving symbiotic partnership and consequent survival in their nutrient-poor environment.

Harii and colleagues hypothesized that the enlargement of the gastric cavity in coral larvae, which may be accompanied by the development of functional, ciliated gastrodermal cells, may enhance acquisition of symbionts by late developmental stages^15^. Indeed, in *Aiptasia* larvae we also find an enlargement of the gastric cavity as a result of the widening of the pharynx and the flattening of the endodermal tissue layer when comparing younger larvae to older larvae. However, such changes do not seem to drastically affect symbiont uptake efficiency of larvae or the total number of symbionts acquired, indicating that the observed changes may simply be developmentally associated and unrelated to symbiosis *per se*. However, we do observe a prominent difference in the spatial distribution of symbiont phagocytosis within the endoderm between younger and older larvae: younger larvae take up symbionts primarily in the aboral region, whereas older larvae efficiently take up symbionts in both the aboral and the oral region of the endoderm. It may be that the distribution of endodermal cells capable of phagocytosing symbionts changes and expands over time, potentially defining predominant symbiont uptake regions within the endoderm. A similar effect has been observed in larvae of the coral *Fungia scutaria*, in which the equatorial region of the endoderm was principally involved in phagocytosis of appropriate symbionts^14^, supporting the idea of functional differences of endodermal cells in corals that may change over time. However, it remains unclear whether the endoderm of anthozoan larvae is differentiated into distinct cell types and, if so, when during development this differentiation occurs. To this end, an important goal of future research is to determine which cellular features (e.g. cilia development, digestive properties, or expression of symbiont-uptake receptors) are responsible for rendering endodermal cells capable of symbiont acquisition and how these relate to larval endodermal development. Additionally, it will be particularly interesting to uncover the molecular mechanisms of when and how cnidarian larvae distinguish between symbionts and inert beads (as a proxy for food particles) (14 and this study, Fig. 3d).

Our analyses presented here generate the framework for such future investigations by providing a thorough description of *Aiptasia* early development in relation to symbiosis establishment as well as essential tools including *in situ* hybridization and confocal microscopy. Together with the recently published *Aiptasia* genome and a robust spawning protocol as important resources^23^,^24^, the field is now well positioned to begin dissecting the mechanisms of symbiont phagocytosis, integration, and maintenance in the cnidarian host cells at the cell and molecular levels, using *Aiptasia* larvae as a model system. The study of endosymbiosis between cnidarians and their symbionts as the foundation of coral reef ecosystems is of broad interest in cell biology as well as of high ecological and evolutionary relevance. Moreover, *Aiptasia* may also help to uncover common principles as well as differences of photosymbiosis, a diverse and complex phenomenon that has been found throughout the tree of life ranging from cnidarians to mollusks (e.g. giant clams and sea slugs) to vertebrates (e.g. salamanders)^47^,^48^,^49^.

## Methods

### *Aiptasia* culture conditions and spawning induction

*Aiptasia* strains CC7 and F003 were cultured and induced to produce larvae as previously described^23^. Larvae were collected and filtered as previously described^23^ and kept in Intellus Ultra Controller Incubators (Model I-36LL4LX, Percival) at 26 °C on a diurnal 12L:12D cycle (12 h light:12 h dark) under white fluorescent bulbs with an intensity of ~20–25 μmol m^−2^ s^−1^ of photosynthetically active radiation (PAR), as measured with an Apogee PAR quantum meter (MQ-200, Apogee).

### *Symbiodinium* culture conditions

Clonal and axenic cultures of *Symbiodinium* strain SSB01^21^ were maintained in IMK medium^50^ at 26 °C and 20–25 μmol m^−2^s^−1^ of photosynthetically active radiation (PAR) as previously described^21^. To determine the approximate algal density of inocula in larval infection experiments, a Neubauer chamber was used for direct microscopic counts.

### Brightfield microscopy of *Aiptasia* embryos and larvae

Embryos and larvae were collected and fixed for 30 min in 3.7% formaldehyde in filter-sterilized artificial seawater (FASW). Specimens were washed three times in PBS-0.2% Triton X-100 (PBT) and then washed into PBS, after which they were mounted in 1:1 glycerol:PBS on glass slides with glass coverslips. Embryos and larvae were imaged with a Nikon Eclipse 80i microscope using Differential Interference Contrast (DIC), a Nikon Plan Fluor 20× dry lens, and a Digital Sight DS-1QM camera (Nikon Instruments).

### *In situ* hybridization of *Aiptasia* embryos and larvae

Probes for *in situ* hybridization of *Aiptasia Forkhead*, *Brachyury*, *β-catenin*, *Chordin*, *Wnt2*, and *OtxA* were designed by using the according *Nematostella vectensis* gene sequences to locate the sequences in the *Aiptasia* genome^24^. Fragments of the genes were amplified from *Aiptasia* CC7^25^ genomic DNA or larval first-strand cDNA via PCR (primer sequences in Table 1). PCR reactions of 50 μl contained 2U Phusion polymerase, 0.1 μM of each primer, 200 μM dNTPs, 1X Phusion HF Buffer (#B0518S, NEB), and 50–150 ng template DNA. Amplification conditions were as follows: initial denaturation at 98 °C for 2 min; 35 cycles of denaturation at 98 °C for 15 s, annealing at 60–63 °C for 30 s, extension at 72 °C for 1 min; final extension at 70 °C for 10 min. Fragments of the expected size were then cloned into the pCRII TOPO-TA Dual Promoter vector (#45-0640, Qiagen) and sequenced with M13F and M13R standard primers to confirm their identity. Digoxygenin-labeled riboprobes were synthesized using the SP6/T7 Transcription Kit (#10999644001, Roche) according to the manufacturer’s instructions.

The following *in situ* hybridization protocol is based on that previously described for *Nematostella*^51^, with modifications. Larvae were fixed for 1 h in 4% formaldehyde in FASW, washed twice in PBT, and then stored in 100% methanol at −20 °C until further use. Fixed larvae were rehydrated by sequential washes in: 100% methanol; 60% methanol/40% PBS-0.1% Tween-20 (PTW); 30% methanol/70% PTW; 100% PTW. Larvae were then permeabilized with 10 μg/ml proteinase K in PTW for 8 min. This was followed by two washes in 2 mg/ml glycine in PTW, one wash in 1% triethanolamine in PTW, and two washes each in 0.3% and 0.6% acetic anhydride/1% triethanolamine in PTW respectively. Larvae were then washed twice in PTW and post-fixed in 4% formaldehyde in PTW for 30 min. After five PTW washes, larvae were transferred into hybridization solution consisting of: 50% formamide, 4X SSC pH 4.5, 50 μg/ml Heparin, 0.25% Tween-20, 1% SDS, 50 μg/ml salmon sperm DNA (#15632-011, Invitrogen). Pre-hybridization was performed first at room temperature (RT) for 10 min, after which the hybridization solution was exchanged and pre-hybridization continued for 1 h at 61 °C. Full hybridization was then carried out at 61 °C for approximately 36 h with 1 ng/ul final probe concentration. Unbound probe was removed by washing twice in hybridization solution, and then larvae were transferred to 2X SSC by sequential washing at 61 °C in the following proportions of hybridization buffer/2X SSC: 100% hybridization solution; 75%/25%; 50%/50%; 25%/75%; 100% 2X SSC. Larvae were then washed twice in 0.05X SSC at 61 °C. Larvae were then transferred to PTW by sequential washing at RT in the following proportions of 0.05X SSC/PTW: 100% 0.05X SSC; 75%/25%; 50%/50%; 25%/75%; 100% PTW. Samples were then blocked in 1X blocking solution (#11096176001, Roche) diluted in maleic acid buffer (0.1 M maleic acid, 0.05 M NaCl) for 30 min at RT. Probe detection was achieved by incubation with an anti-DIG alkaline-phosphatase-conjugated antibody (#11093274910, Roche) diluted 1:5000 in blocking solution overnight at 4 °C. After 10 washes in PBT, larvae were washed twice in AP buffer (0.1 M Tris-HCl pH 9.5, 0.1 M NaCl, 0.1% Tween 20) without MgCl_2_ and twice in AP buffer with 0.05 M MgCl_2_. Probe detection was performed with NBT/BCIP (#11681451001, Roche) diluted 1:50 in buffer (0.1 M Tris-HCl pH 9.5, 0.1 M NaCl). Detection was stopped with several rinses in 100% ethanol, and larvae were mounted in 1:1 glycerol:PBS on glass slides with glass coverslips Specimens were imaged with a Nikon Eclipse 80i microscope using Differential Interference Contrast (DIC), a Nikon Plan Fluor 20× dry lens, and a Digital Sight DS-U1 color camera (Nikon Instruments).

### Symbiosis establishment in *Aiptasia* larvae

For infections, larvae were counted as previously described^23^ and distributed in 6-well plates with 300–500 larvae in 5 ml FASW per well. Infection with *Symbiodinium* strain SSB01^21^ was performed by adding algae to each well at a final concentration of 1,000, 10,000, or 100,000 algal cells/ml, as indicated in the text. After addition of algae, each well was mixed by gently pipetting up and down. Infection experiments with inert polystyrene fluorescent beads (#C36950, Life Technologies) were carried out identically; FASW was used as a negative control. Larvae were then fixed for 1 h in 4% formaldehyde in FASW, washed three times in PBT, washed into PBS, and then mounted in 1:1 glycerol:PBS on glass slides with glass coverslips. Slides were analyzed using a Nikon Eclipse 80i microscope with a Nikon Plan Fluor 20× dry lens. A minimum of 70 larvae per slide were scored per condition per time-point.

### Confocal microscopy of *Aiptasia* embryos and larvae

For confocal imaging, embryos and larvae were fixed in 3.7% formaldehyde in FASW for 30 min, washed three times in PBT, and washed once in PBS. Approximately 50–100 larvae were transferred to PCR tubes and permeabilized with 10 μg/ml proteinase K in PBS for 8 min. Permeabilization was stopped by washing twice for 5 min in 2 mg/ml glycine in PBT. Larvae were post-fixed in 3.7% formaldehyde in PBT, then washed twice for 10 min in PBT and twice for 10 min in PBS. Larvae were incubated with AlexaFluor-488 phalloidin (#A12379, Invitrogen) diluted 1:300 in PBS for 1 h at RT on a rotor (Intelli mixer #7-0045, NeoLab) at a speed of 20 rpm. Larvae were then washed twice for 15 min in PBT and incubated with 10 μg/ml Hoechst in buffer (Tris-buffered saline, pH 7.4, 0.1% Triton X-100, 2% bovine serum albumin, 0.1% sodium azide) for 15 min. Final washes were carried out in PBT three times each for 15 min. Samples were mounted in 87% glycerol in PBS containing 2.5 mg/ml DABCO (1,4-Diazabicyclo[2.2.2]octan, #D27802, Sigma Aldrich). Images of embryos and larvae in Figs. 2 and 3f were acquired using a Nikon A1 confocal microscope with a Nikon Plan Fluor 40× oil immersion objective and Nikon Elements Software. Images of intracellular algae in Fig. 3c were acquired using a Leica TCS SP5II confocal microscope with a Leica HCX PL APO lambda blue 63.0 × 2.10 UV water immersion objective and Leica Application Suite Advanced Fluorescence software. Image processing and maximum projections of Z-stacks was performed using Fiji^52^.

## Additional Information

**How to cite this article**: Bucher, M. *et al.* Development and symbiosis establishment in the cnidarian endosymbiosis model *Aiptasia* sp. *Sci. Rep.* **6**, 19867; doi: 10.1038/srep19867 (2016).

## Figures and Tables

**Table 1:** Primers used to generate *in situ* hybridization probes.

*Brachyury* F	5´ - AACCATATCCTTCAAGCCGCA - 3´
*Brachyury* R	5´ - AGATCCGCGCGCTTGTAATA - 3´
*Forkhead* F	5´ - AAGGCGCGCCGATCCCTCGCAAAACCCTCA - 3´
*Forkhead* R	5´ - AATTAATTAAGCAATTCGCCGCTGTAAACA - 3´
*β-catenin* F	5´ - AAGGCGCGCCTGGACACTGCGTAACCTGTC - 3´
*β-catenin* R	5´ - TTTTAATTAAGTTGTGTCGCGTTTTCAGCT - 3´
*Chordin* F	5´ - AAGGCGCGCCTCAGGCGCCATTCACAGATT - 3´
*Chordin* R	5´ - TTTTAATTAACACTTGGGTACGTCACGACA - 3´
*Wnt2* F	5´ - AAGGCGCGCCGGTTGAATTCCAAATGAATAACAA - 3´
*Wnt2* R	5´ - TTTTAATTAACAACACCAATAAAACTTACAGTAGCA - 3´
*OtxA* F	5´ - AAGGCGCGCCTGACTCCTCCAAACATTGATTTCT - 3´
*OtxA* R	5´ - TTTTAATTAAGGGATTGCCTATCTGTGACGA - 3´
